# Endoplasmic Reticulum Protein Quality Control Failure in Myelin Disorders

**DOI:** 10.3389/fnmol.2016.00162

**Published:** 2017-01-04

**Authors:** Vera G. Volpi, Thierry Touvier, Maurizio D'Antonio

**Affiliations:** Biology of Myelin Unit, Division of Genetics and Cell Biology, San Raffaele Scientific Institute, DIBITMilan, Italy

**Keywords:** protein folding, ERAD, UPR, proteostasis failure, oligodendrocyte, Schwann cell, dysmyelination

## Abstract

Reaching the correct three-dimensional structure is crucial for the proper function of a protein. The endoplasmic reticulum (ER) is the organelle where secreted and transmembrane proteins are synthesized and folded. To guarantee high fidelity of protein synthesis and maturation in the ER, cells have evolved ER-protein quality control (ERQC) systems, which assist protein folding and promptly degrade aberrant gene products. Only correctly folded proteins that pass ERQC checkpoints are allowed to exit the ER and reach their final destination. Misfolded glycoproteins are detected and targeted for degradation by the proteasome in a process known as endoplasmic reticulum-associated degradation (ERAD). The excess of unstructured proteins in the ER triggers an adaptive signal transduction pathway, called unfolded protein response (UPR), which in turn potentiates ERQC activities in order to reduce the levels of aberrant molecules. When the situation cannot be restored, the UPR drives cells to apoptosis. Myelin-forming cells of the central and peripheral nervous system (oligodendrocytes and Schwann cells) synthesize a large amount of myelin proteins and lipids and therefore are particularly susceptible to ERQC failure. Indeed, deficits in ERQC and activation of ER stress/UPR have been implicated in several myelin disorders, such as Pelizaeus-Merzbacher and Krabbe leucodystrophies, vanishing white matter disease and Charcot-Marie-Tooth neuropathies. Here we discuss recent evidence underlying the importance of proper ERQC functions in genetic disorders of myelinating glia.

## Introduction

### Protein folding and ER-associated degradation (ERAD)

**Figure 1:**
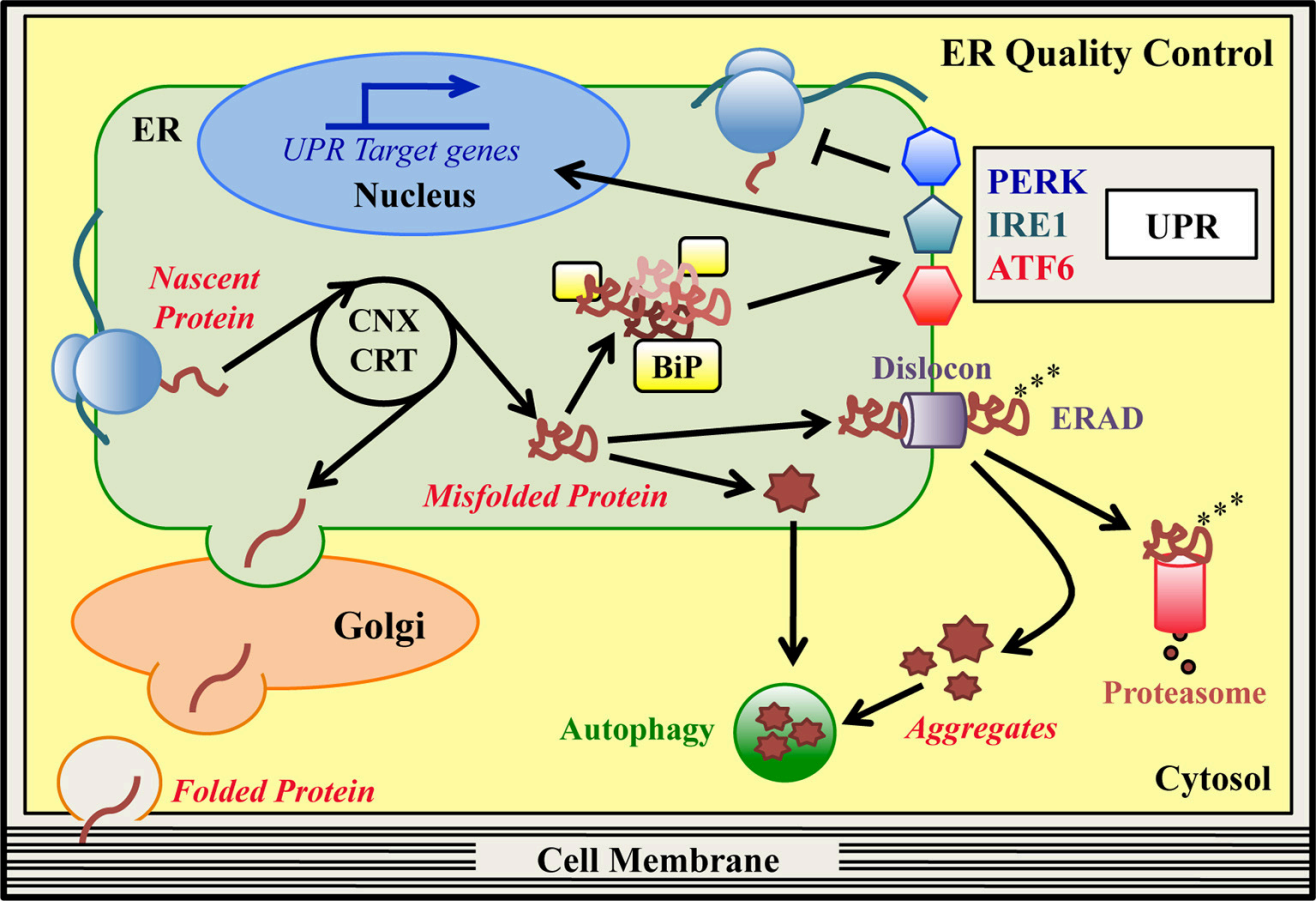
**ER quality control (ERQC) pathways**. In the ER specialized protein quality control (ERQC) pathways assist the synthesis, folding and maturation of proteins destined for secretion or insertion into the cell membrane. ER-synthesized proteins emerge from ER-bound ribosomes and reach their native structure, guided by ER-resident chaperones, such as CNX, CRT and BiP. Proteins that fold correctly transit to the Golgi stacks to complete their maturation and are then delivered to their final destination. Proteins that are unable to reach their native conformation, designated as “terminally misfolded,” are eliminated to counter potential proteo-toxicity. The main degradation system in the ER is the ER-associated degradation (ERAD) pathway. In this pathway, misfolded ERAD substrates are recognized by specialized ER lectins and dislocated from the ER to the cytosol by dedicated ERAD dislocation complexes (dislocons). In concomitance with the dislocation reaction, ubiquitinating enzymes attach ubiquitin moieties to the aberrant polypeptide chains as a signal for proteasomal recognition. Once in the cytosol, polyubiquitinated ERAD substrates are detected by the proteasome and degraded. Hence, basal ERQC activities cooperate to finely tune the balance between protein production and clearance. When the ERAD/proteasome system is engulfed and/or proteins form intracellular aggregates, autophagy may be activated as an alternative degradation system. Mutations that hamper the achievement of a proper protein structure can produce an excess of misfolded proteins in the ER, which exhausts the basal ERQC capacities generating ER stress. Under these conditions, BiP detects the excess of misfolded proteins and initiates the unfolded protein response (UPR) cascade, which is mediated by the PERK, IRE1, and ATF6 signaling pathways. The final goal of the UPR is to reduce the amount of misfolded proteins in the ER and re-establish ER-homeostasis. PERK (via phosphorylation of the translation initiation factor eIF2α) attenuates global protein synthesis. Concomitantly, activated IRE1/XBP1 and ATF6 pathways upregulate ERAD, ER-chaperones and autophagy genes, and genes associated with amino acid and lipid metabolism. If the ER stress cannot be resolved, the UPR itself can drive cells to apoptosis.

The endoplasmic reticulum (ER) is a cellular compartment committed to the synthesis of secreted and transmembrane proteins, lipid production and calcium storage. Newly synthesized proteins co-translationally enter the ER, where they promptly undergo processes of folding and post-translational modification to achieve their native conformation (Hebert and Molinari, 2007; Braakman and Hebert, 2013). Reaching a proper structure is crucial for all proteins, since structure and function are strictly interconnected. Indeed, aberrant conformations can result in nonfunctional or toxic proteins, which can compromise cell viability. Specialized surveillance systems, the ER-protein quality control (ERQC) pathways, cooperate to guarantee high fidelity of protein synthesis and maturation to preserve ER homeostasis (Figure 1; Ellgaard and Helenius, 2003; Bergmann et al., 2016). These pathways discriminate between correct or aberrant gene products, so that only structured and fully functional proteins are delivered to their final destination.

A cohort of ER-resident chaperones and folding enzymes co- and post-translationally facilitate the generation of the emerging polypeptides (Ni and Lee, 2007; Braakman and Hebert, 2013). Classical ER chaperones, such as immunoglobulin heavy chain-binding protein/glucose regulated protein 78 (BiP/GRP78) and glucose regulated protein 94 (GRP94), support the spontaneous folding of proteins acting as guidance molecules (Ni and Lee, 2007). Numerous folding enzymes, instead, accelerate the folding reaction and catalyze disulfide bonds oxidoreduction, glycans addition to asparagine residues (N-glycosylation) and protein oligomerization (Hebert and Molinari, 2007; Braakman and Hebert, 2013). Fundamental ERQC factors are the lectin chaperones calnexin (CNX) and calreticulin (CRT), which assist the folding of ER-synthesized glycoproteins (Figure 1; Lamriben et al., 2016). In the CNX/CRT cycle, the modification of the glycan portion attached to the unfolded glycoproteins drives the alternate dissociation and re-binding of these proteins to the lectins, until they reach their proper structure. Then, correctly folded glycoproteins exit the cycle and travel along the secretory pathway toward their destination (Lamriben et al., 2016). Instead, terminally misfolded glycoproteins are targeted to ER-associated degradation (ERAD) for their disposal (Guerriero and Brodsky, 2012; Olzmann et al., 2013; Xu and Ng, 2015; Lamriben et al., 2016). In ERAD of glycoproteins, specialized ER mannosidases remove mannose residues from the misfolded molecules, so that they can be recognized by mannose-specific lectins and shuttled from the ER to the cytosol. Importantly, BiP and GRP94 have been found to interact with these ER lectins, suggesting a role for the two chaperones in ERAD (Xu and Ng, 2015). Following substrate recognition, a dislocation reaction takes place, favored by multi-protein complexes (called dislocons) that span the ER membranes (Olzmann et al., 2013). In mammals, some key dislocon components are the scaffold protein Suppressor/Enhancer of Lin-12-like (Sel1L), the ER dislocation factors Derlin1, -2 and -3, and several ubiquitinating enzymes, such as HMG-CoA Reductase Degradation protein 1 (HRD1) and gp78/autocrine motility factor receptor (AMFR). Moreover, the cytosolic valosin containing protein (VCP)/p97 ATPase is thought to provide the energy required for the dislocation reaction (Guerriero and Brodsky, 2012; Olzmann et al., 2013). During dislocation, ubiquitinating enzymes add ubiquitin moieties to the aberrant polypeptide chains. This protein modification targets the misfolded molecules to degradation by the proteasome in the cytosol (Christianson and Ye, 2014; Schmidt and Finley, 2014; Livneh et al., 2016). How misfolded non-glycosylated proteins are folded, recognized and dislocated by the ERAD machinery is less characterized (Bergmann et al., 2016). The ERAD and proteasome machineries, thus, exert a pivotal role in the quality control and clearance of specific ER clients. When the ubiquitin-proteasome clearing system is overwhelmed and/or misfolded proteins form insoluble aggregates, the cells may get rid of aberrant proteins by activating autophagy (Figure 1; Hyttinen et al., 2014; Cohen-Kaplan et al., 2016).

### The unfolded protein response (UPR)

Accumulation of unstructured/misfolded proteins that exhausts the basal quality control capacity of the ER often results in ER stress (Ron and Walter, 2007; Wang and Kaufman, 2016). This imbalance is frequently associated with several disease states, such as conformational disorders, neurodegeneration and cancer, but also to those physiological processes in which extensive protein production is required (Wang and Kaufman, 2016). To deal with the ER overloading, cells activate the unfolded protein response (UPR) (Figure 1; Ron and Walter, 2007; Hetz et al., 2015). The UPR is a pro-survival strategy aimed at reducing the excess of misfolded proteins in the ER and re-establishing ER homeostasis, in particular by stimulating ERQC activities. The ER chaperone BiP is also involved in sensing the amount of misfolded proteins in the ER and initiating the UPR cascade (Ron and Walter, 2007). Three ER stress sensors transduce the UPR pathways: protein-kinase RNA-like endoplasmic reticulum kinase (PERK), inositol-requiring enzyme 1 (IRE1) and activating transcription factor 6 (ATF6) (Ron and Walter, 2007; Hetz et al., 2015). In normal conditions, BiP binds to IRE1 and PERK, keeping them inactive. Under ER stress, BiP preferentially binds the misfolded molecules and dissociates from the two transducers allowing their activation by oligomerization and autophosphorylation (Ron and Walter, 2007; Hetz et al., 2015). Once activated, PERK phosphorylates the translation initiation factor eIF2α (P-eIF2α), transiently attenuating general protein synthesis (Figure 1). Paradoxically, this is accompanied by increased translation of the mRNA for the transcription factor ATF4, that induces the expression of chaperones, antioxidant-, amino acid metabolism- and autophagy-related genes, but also anti-survival genes, such as *CHOP/Ddit3* (Ron and Walter, 2007; Hetz et al., 2015). CHOP, a transcription factor, in turn upregulates GADD34/PPP1R15a, a regulatory subunit of the phosphatases complex in charge of dephosphorylating P-eIF2α and as such restarting translation (Novoa et al., 2001). Importantly, upon nutrient starvation, UV irradiation and viral infections, kinases other than PERK catalyze eIF2α phosphorylation, without the concomitant activation of the IRE1 and ATF6 pathways. In this way the phosphorylation of eIF2α “integrates” a plethora of different stresses, in a response known as the integrated stress response (ISR) (Donnelly et al., 2013; Pakos-Zebrucka et al., 2016). The UPR-specific IRE1 and ATF6 pathways, instead, converge on the transcriptional upregulation of ER folding chaperones and enzymes, protein export, lipid metabolism and ERAD genes (Figure 1; Acosta-Alvear et al., 2007; Yamamoto et al., 2007; Shoulders et al., 2013). To orchestrate this program, two transcription factors are recruited. One is spliced X-box binding protein-1 (XBP1s), which is generated via the IRE1-mediated unconventional splicing of XBP1 mRNA; the other is the cleaved cytosolic domain of ATF6 (ATF6-f fragment), produced by the S1P/S2P cleavage of the full length ATF6 (Ron and Walter, 2007; Hetz et al., 2015). More recently, several other mechanisms have been proposed to contribute in reducing the protein flux into the ER upon ER stress. These include the regulated IRE1-dependent decay (RIDD) of ER localized mRNAs (Hollien and Weissman, 2006), a selective mRNA release from the site of translation (Reid et al., 2014) and a pre-emptive quality control degradation system (Kadowaki et al., 2015). When the adaptive UPR fails and ER stress persists, the response may switch to a terminal maladaptive phase, leading damaged cells to apoptosis (Ron and Walter, 2007; Hetz et al., 2015).

Proficient secretory cells, in charge of abundant protein production, require highly efficient ERQC systems to survive under sustained ER stress. In the central and peripheral nervous systems (CNS and PNS), oligodendrocytes and Schwann cells are the glial cells specialized in the formation of myelin (Monk et al., 2015; Hughes and Appel, 2016). Myelin is a multi-layer membrane that enwraps the axons to favor the saltatory conduction of the nerve impulse, provide axonal protection, allow axon-glia communication and mediate signaling with the extracellular environment (Jessen and Mirsky, 2005; Nave and Werner, 2014; Bercury and Macklin, 2015). In mammals, myelin formation is a post-natal event that requires the production of large amounts of membrane components, such as lipids and myelin-specific proteins. After the completion of myelin biogenesis, Schwann cells and oligodendrocytes are committed to preserve long-term myelin stability and functionality. Defective myelin development (dysmyelination) or myelin instability and its consequent degeneration (demyelination) often result in mild-to-very severe neurological and neuromuscular disorders. Notably, animal models carrying mutations in key ERQC factors display CNS and PNS disease phenotypes. In addition, ERQC failure, with subsequent ER stress and UPR activation, has been associated with several genetic disorders of myelinating glia. As such, the modulation of ERQC functions is emerging as a promising target for the treatment of ER stress-related myelin disorders. All of these aspects will be here overviewed and discussed.

### ERQC factors and dysmyelination

As a central site for the biosynthesis of proteins and lipids, the ER is the organelle where many myelin components are produced. Several transmembrane myelin proteins of both the CNS and the PNS are synthesized on ER-bound ribosomes, post-translationally modified and delivered to myelin within vesicles. In the myelin sheath they often function as adhesion molecules, connecting adjacent wraps of membrane (Trapp et al., 1981, 1995; D'Urso et al., 1990; Brunden, 1992; Pareek et al., 1993, 1997; White and Krämer-Albers, 2014). It should come as no surprise then that the genetic ablation of factors involved in the folding and maturation of these proteins results in severe dysmyelinating phenotypes.

As previously discussed, BiP, member of the heat shock protein (HSP) family, participates in various ER functions (Hendershot, 2004; Ron and Walter, 2007; Dudek et al., 2009). Full BiP^−/−^ mice die at the embryonic stage, whereas BiP^+/−^ mice develop normally (Zhu and Lee, 2015). A number of tissue-specific BiP knock-out mice revealed that BiP contributes in maintaining homeostasis of different organs, including the liver, the adipose tissue and the hematopoietic system (Zhu and Lee, 2015). Different BiP mutant mouse models, such as hypomorphic mutants, knock-in and conditional knock-out mice, display neuronal death and degeneration, reduction in brain size, cortical dysplasia, defective axon outgrowth and severe CNS and PNS myelin abnormalities (Mimura et al., 2008; Wang et al., 2010; Favero et al., 2013; Jin et al., 2014; Hussien et al., 2015). In particular, the conditional ablation of BiP either in oligodendrocytes or in Schwann cells strongly affects myelin formation and maintenance, resulting in cell loss, severe developmental hypomyelination (thinner myelin), demyelination and motor defects. Of note, the oligodendrocyte-specific ablation of BiP results in ER stress and UPR activation, with upregulation of CHOP and increased oligodendrocytes apoptosis in some regions of the brain. Finally, heterozygous deletion of BiP in experimental autoimmune encephalomyelitis (EAE) animals, a model of multiple sclerosis, exacerbates the disease phenotype, with increased CHOP expression and oligodendrocytes death. Altogether these observations indicate that BiP is important in myelin physiology and is involved in countering inflammatory demyelination (Hussien et al., 2015).

**Figure 2:**
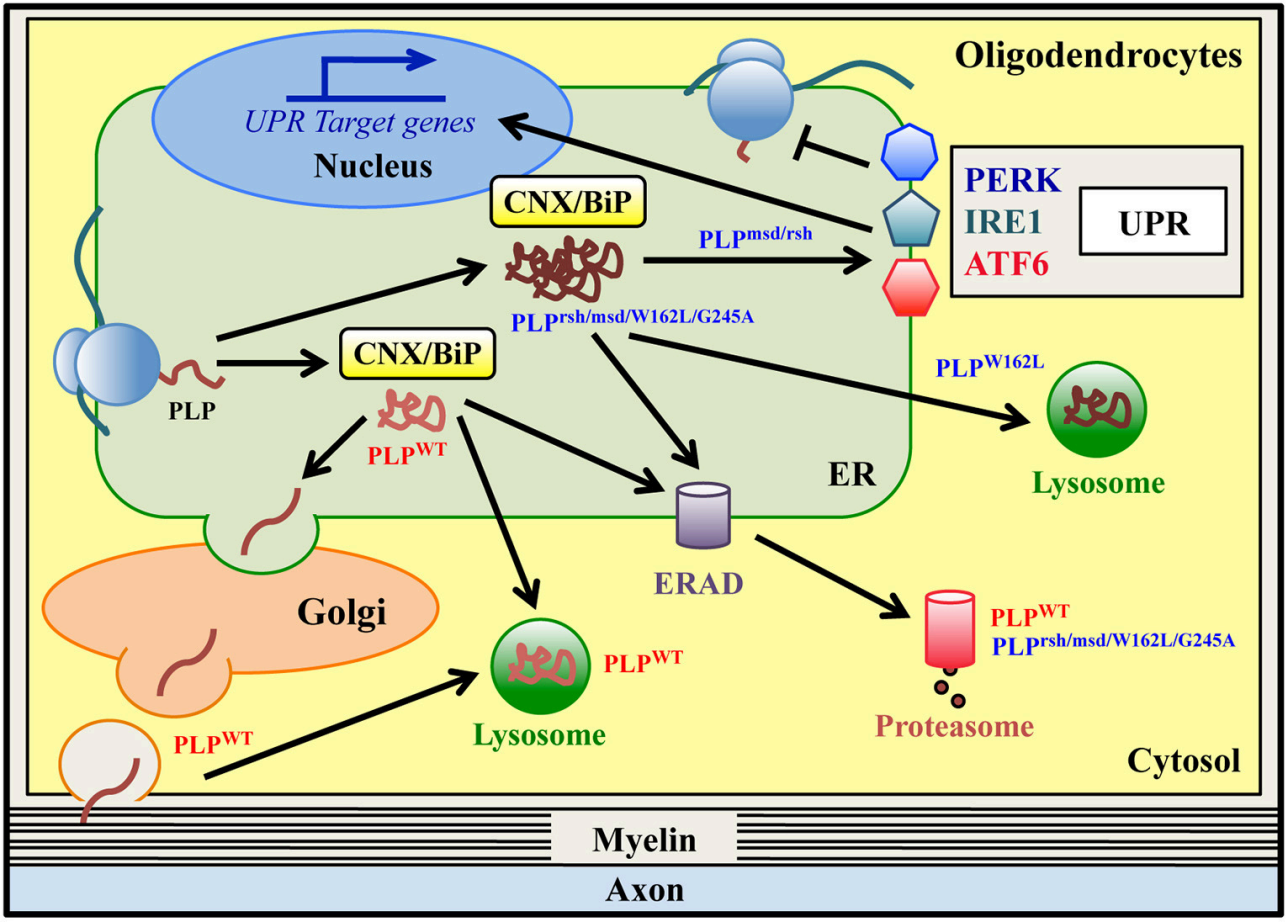
**ER quality control of wild-type and mutant PLPs in oligodendrocytes**. Proteolipid Protein (PLP) is the most abundant protein of CNS myelin. Wild type PLP protein (PLP^WT^) is synthesized in the ER and folded via transient and glycan-independent interaction with BiP and CNX. It then moves to the Golgi, where it associates with lipid rafts, and finally it is targeted to myelin. PLP^WT^ is normally degraded via the ERAD/proteasome system and/or lysosomes. Mutations in PLP result in Pelizaeus-Merzbacher Disease (PMD), a genetic leucodystrophy that affects CNS myelin in both humans and related-mouse models. Some PLP mutants (PLP^rsh/msd/W162L/G245A^) are misfolded proteins that accumulate in the ER, stably interacting with the ER retention factors BiP and CNX. All of these mutants are degraded via the ERAD/proteasome system, albeit with a different rate of degradation. The PLP^W162L^ mutant can also be disposed of by lysosomes. The PLP^msd^ and PLP^rsh^, but not the PLP^W162L/G245A^ variants, induce the UPR response *in vitro* and *in vivo*.

**Figure 3:**
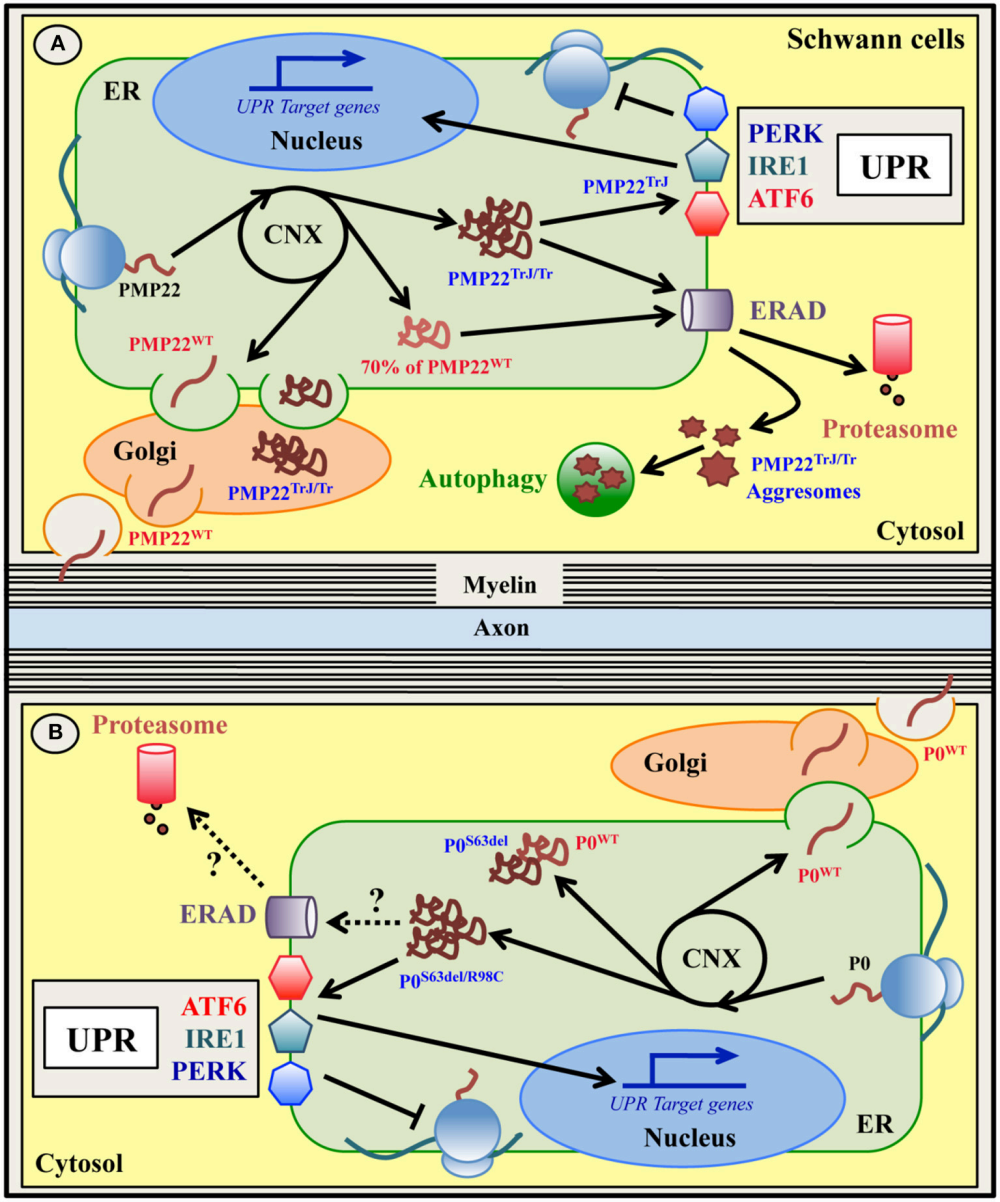
**ER quality control of wild type and mutant PMP22 (A)** and P0 **(B)** glycoproteins in Schwann cells. Peripheral myelin protein 22 KDa (PMP22) and myelin protein zero (P0) are glycoproteins expressed by myelinating Schwann cells in the PNS and normally located in compact myelin. Both PMP22 and P0 are synthesized on ER-bound ribosomes, folded via transient interaction with CNX, N-glycosylated and delivered to PNS myelin after transition within the Golgi stacks **(A,B)**. Up to 70% of newly synthesized wild type PMP22 (PMP22^WT^) is degraded by the proteasome **(A)**. Mutations in PMP22 and P0 are associated with different forms of Charcot-Marie-Tooth (CMT) neuropathy in both humans and mice. A subset of these mutations, such as the Trembler (G150D)/Trembler-J (L16P) mutations in PMP22 and the S63del and R98C mutations in P0, produce misfolded proteins that accumulate in the ER-Golgi **(A,B)**. Both PMP22^TrJ^ and PMP22^Tr^ variants have been reported to engulf the ERAD/proteasome system and form cytoplasmic aggresomes, partially cleared by autophagy **(A)**. Oppositely, neither the P0^S63del^ nor the P0^R98C^ proteins form intracellular aggregates **(B)**. Whether ERAD and/or autophagy are involved in the disposal of these P0 mutants demands investigation. Notably, both the P0^S63del/R98C^ and the PMP22^TrJ^ variants (but not the PMP22^Tr^ protein), activate the UPR response *in vitro* and *in vivo* **(A,B)**. The P0^S63del^ protein (and the PMP22 mutants) also appears to exert a dominant negative effect on its corresponding wild type protein, trapping it in the ER and reducing its level in the myelin membrane **(B)**.

The ER lectin CNX has also been shown to be important for the physiology of both the CNS and PNS. In fact, CNX, but not CRT, appears to play a role in the folding of peripheral myelin protein 22 (PMP22) and myelin protein zero (P0) in the PNS (**Figure 3**; Dickson et al., 2002; Jung et al., 2011) and in the quality control of wild-type and misfolded proteolipid protein (PLP) (Figure 2) and myelin oligodendrocytes glycoprotein (MOG) in the CNS (Swanton et al., 2003; Jung and Michalak, 2011; Jung et al., 2015). In mice, the complete lack of CNX, but not CRT, is viable, but CNX^−/−^ mice display severe motor impairment and myelin defects in both CNS and PNS (Mesaeli et al., 1999; Denzel et al., 2002; Kraus et al., 2010). In cultured CNX-deficient cells, both the folding and the adhesive function of GFP-tagged P0 and PMP22 glycoproteins are impaired (Jung et al., 2011). Thus, in the PNS, the dysmyelination might be related to the lack of proper quality control of wild-type PMP22 and P0 proteins (Denzel et al., 2002; Kraus et al., 2010; Kraus and Michalak, 2011). In addition to CNX, ERp57, an ER-resident disulfide isomerase, has been implicated in the folding of the disulfide-bond-containing protein P0, but not in the folding of MOG, suggesting that different ERQC factors are involved in the maturation and post-translational modification of different myelin proteins (Jung et al., 2011; Jung and Michalak, 2011).

In light of these observations, it will be of great interest to further investigate how other ERQC components, for example members of the ERAD/proteasome machineries known to participate in the degradation of wild-type and mutant myelin proteins (Pareek et al., 1993, 1997; Hara et al., 2014; Lossos et al., 2015), are involved in myelinogenesis, myelin maintenance and disease.

## ER protein quality control and myelin diseases of the CNS

### ERQC and pelizaeus-merzbacher disease

Pelizaeus-Merzbacher disease (PMD) is a rare inherited leukodystrophy affecting CNS myelin. The signs of PMD may vary widely from case to case. Patients affected by classical PMD exhibit lower limb spasticity and have a relatively normal life span, whereas patients with the connatal form never learn to talk or walk and have a markedly shortened life span (Hobson and Garbern, 2012). PMD patients have a diffuse pattern of hypomyelination in the CNS, but in most severe cases a nearly complete absence of myelin and a profound loss of oligodendrocytes are observed. PMD is caused by a variety of missense, duplication and deletion mutations in the *PLP1* gene (Garbern, 2005). *PLP1* is highly expressed in oligodendrocytes and encodes two tetraspan proteins, proteolipid protein (PLP) and its splice variant DM20. These are the most abundant proteins in CNS myelin and play a structural role in the formation and maintenance of the myelin sheaths (Greer and Lees, 2002).

Several mouse models are currently available to study both PLP function and the pathogenesis of PMD. Notably, some missense mutations found in patients are also naturally occurring in mice. For example, the A242V amino acid substitution, that causes a severe form of PMD in humans, is found in the myelin synthesis-deficient (*msd*) mouse, whereas the I186T mutation, which results in a milder disease in humans, is found in the rumpshacker (*rsh*) mouse (Duncan, 2005). Another mutant, the jimpy (*jp*) mouse, has been extensively used as a PMD model, even if the same mutation is not found in humans (Baumann and Pham-Dinh, 2001). The pathogenetic mechanism underlying severe PMD caused by missense mutations is likely a gain of toxic function of PLP mutants. In fact, individuals with null PLP mutations present a less severe form of the disease (Garbern et al., 1997), and similarly, *PLP1* knock-out mice display a milder phenotype as compared to *msd* or *rsh* mice (Klugmann et al., 1997). Finally, in *jp* mice the dysmyelination and oligodendrocyte death are not corrected by transgene-driven expression of wild-type PLP and DM20 (Nadon et al., 1994; Schneider et al., 1995).

Wild-type PLP (PLP^WT^) is synthesized in the ER and subsequently transported to the Golgi complex, where it associates with myelin lipids into rafts before reaching the cell surface (Lee, 2001). Most of the PLP^WT^ is degraded via the lysosomal pathway, although a minor fraction may also be degraded by the proteasome (Figure 2; Roboti et al., 2009). Interestingly, data obtained in various *in vitro* and *in vivo* PMD models demonstrate that the PLP mutant proteins do not reach the cell surface but accumulate in the ER (Gow et al., 1994, 1998; Gow and Lazzarini, 1996; Krämer-Albers et al., 2006; Roboti et al., 2009; Numasawa-Kuroiwa et al., 2014) and studies using conformationally-sensitive antibodies show that these point mutations cause PLP misfolding (Jung et al., 1996; Southwood and Gow, 2001). Remarkably, mutations inducing a complete arrest of PLP/DM20 trafficking in the ER are associated with the most severe form of the disease and with oligodendrocyte death (connatal PMD), whereas mutations resulting in a partial block of PLP and/or DM20 trafficking are associated with milder disease phenotype (classical PMD) (Gow and Lazzarini, 1996; Southwood and Gow, 2001). Several mechanisms have been proposed to explain the ER retention of misfolded PLP mutants. Abnormal intramolecular cross-links formed by exposure of unpaired cysteine residues might contribute to mutant PLP misfolding and ER retention (Dhaunchak and Nave, 2007). Moreover, the A242V mutant of PLP (PLP^msd^) stably interacts with the ER chaperones BiP and CNX, which may act as ER retention factors (Figure 2; Swanton et al., 2003). In particular, CNX stably binds to a transmembrane domain of mutant PLP^msd^ (but not to wild-type PLP) and retards its degradation (Swanton et al., 2003). The extent of mutant PLP accumulation in the ER may also correlate to its degradation rate. It has been observed that ER-retained PMD mutants, for example PLP^msd^, PLP^W162L^, and PLP^G245A^ variants, are degraded by the proteasome via ERAD (Figure 2 and Table 1; Krämer-Albers et al., 2006; McLaughlin et al., 2006; Roboti et al., 2009). Notably, the PLP^msd^ variant, that causes severe PMD, was found to be more stable than the PLP^W162L^ and PLP^G245A^ mutants, which cause a milder disease (Roboti et al., 2009). This suggests that higher resistance to ERAD may contribute in determining a more severe manifestation by increasing the accumulation of mutant PLP^msd^ in the ER (Roboti et al., 2009). The ER retention of misfolded PLP proteins can also induce the UPR, as observed in some PMD models including transfected cells, mouse models and in post-mortem specimen from a PMD patient (Hudson and Nadon, 1992; Southwood and Gow, 2001; Southwood et al., 2002, 2013; McLaughlin et al., 2007; Roboti et al., 2009). Interestingly, Roboti et al. reported that the “aggressive” PLP^msd^ mutant appears to induce a strong increase of XBP1s and CHOP mRNA levels, whereas the expression of the less “aggressive” mutants (PLP^W162L^ and PLP^G245A^) fails to upregulate these UPR markers (Roboti et al., 2009), implying a possible correlation between UPR activation and disease severity. However, such a clear correlation is not observed in other studies, where the expression of the PLP^rsh^ variant (I186T mutation), associated with a mild disease phenotype, is sufficient to activate the UPR (Southwood et al., 2002; McLaughlin et al., 2006). Thus, the molecular mechanisms explaining the difference between mild and severe PLP mutants still remain partially unclear. Interestingly, a recent study reported that a proinflammatory environment might be an additional factor that participate to PMD pathogenesis and may contribute to disease severity (Southwood et al., 2013). The pathogenetic mechanism proposed in PMD may also apply to disorders clinically related to PMD caused by mutations in myelin genes other than *PLP1*. Indeed, a missense mutation in the myelin associated glycoprotein (*MAG*) gene, which induces a PMD-like disorder, affects MAG folding and prevents its transport to the cell surface. The mutant protein accumulates in the ER, interacts with BiP and CNX and is subjected to ERAD (Table 1; Lossos et al., 2015). This suggests that the ER retention may represent a common pathogenetic mechanism in PMD and PMD-like disorders.

**Table 1:** **Evidence of ERQC involvement and failure in genetic disorders of myelinating glia caused by ER-retained proteins**.

**CNS**								
**Protein**	**Mutation**	**Disease**	**BiP/CNX Interaction**	**Degradation pathway**	**Aggregates formation**	**Activated UPR markers**	**Effect of CHOP ablation**	**OL Apoptosis**
**PLP**	A242V (PLP^msd^)	PMD (Connatal)	CNX/BiP	ERAD/ Proteasome	No	CHOP, BiP, XBP1s, ATF3, caspase 12	N.D.	+++
	I186T (PLP^rsh^)	PMD (Classical)	N.D.	ERAD/ Proteasome	No	CHOP, BiP, XBP1s, ATF3, caspase 12	PMD Worsening	+ or +++^*^
	G245A (PLP^G245A^)	PMD (Classical)	N.D.	ERAD/ Proteasome	No	No	N.D.	N.D.
	W162L (PLP^W162L^)	PMD (Classical)	N.D.	ERAD/ Proteasome/ Lysosome	No	No	N.D.	N.D.
**MAG**	S133R (MAG^S133R^)	PMD-like	CNX/BiP	ERAD/ Proteasome	N.D.	N.D.	N.D.	N.D.
**GALC**	T529M (GALC^T529M^)	KD	N.D.	N.D	N.D	N.D	N.D	N.D.
	G286D (GALC^G286D^)	KD	N.D.	N.D	N.D	N.D	N.D	N.D.
**PNS**								
**Protein**	**Mutation**	**Disease**	**BiP/CNX Interaction**	**Degradation pathway**	**Aggregates formation**	**Activated UPR markers**	**Effect of CHOP ablation**	**SC Apoptosis**
**PMP22**	L16P (PMP22^TrJ^)	CMT1E	CNX/ Rer1^**^	ERAD/ Proteasome/ Autophagy	Yes	BiP, CHOP, XBP1s, ATF3	N.D.	+
	G150D (PMP22^Tr^)	DSS	CNX	ERAD/ Proteasome	Yes	N.D.	N.D.	+
**P0**	S63del (P0^S63del^)	CMT1B	BiP?	N.D.	No	CHOP, BiP, XBP1s, ATF6-f, P-eIF2a, ATF4, GADD34, ATF3	CMT1B Amelioration	+
	R98C (P0^R98C^)	DSS	N.D.	N.D.	No	BiP, CHOP, XBP1s, ATF6-f	None	+

*OL, Oligodendrocytes; SC, Schwann cell; N.D., Not Determined*;

* *, Depending on genetic background*;

** *, Golgi retention*.

One crucial question for understanding PMD pathogenesis is why protein accumulation in the ER and the subsequent UPR activation is deleterious for oligodendrocyte function or survival. Few studies have tried to manipulate supposed pro-apoptotic factors of the UPR, such as CHOP, ATF3 or Caspase12 in PMD mouse models and led to rather surprising conclusions. While ablation of ATF3 or Caspase12 has only minor effect on disease severity both in *msd* and *rsh* mice (Sharma and Gow, 2007; Sharma et al., 2007), the genetic ablation of CHOP dramatically worsens the disease phenotype in *rsh* mice (Southwood et al., 2002). In fact, *rsh*/Chop^−/−^ mice die as early as 5 weeks of age and exhibit frequent seizures following handling or sudden noises as compared to *rsh* mice that have a normal life span. This extreme exacerbation of the phenotype is accompanied by an increase of oligodendrocyte apoptosis in the spinal cord and in the optic nerve (Southwood et al., 2002). These data indicate that CHOP activation attenuates PMD severity and protects oligodendrocytes from apoptosis, supporting the idea that the CHOP arm of the UPR has an adaptive role in PMD. The mechanisms underlying oligodendrocytes death in these models still remain elusive. This also opens questions on the role of CHOP in myelin disorders. Indeed numerous studies carried out in the CNS and the PNS (see below) do not support a role for CHOP in apoptosis, but suggest it should be viewed as a mediator of responses to stress (Gow and Wrabetz, 2009). Consistently, a recent study shows that CHOP overexpression in oligodendrocytes and Schwann cells of wild-type, *msd* and *rsh* mice does not influence animal life span or oligodendrocytes apoptosis (Southwood et al., 2016). Interestingly, the authors report that only half of myelinating oligodendrocytes from *rsh* mice expresses CHOP and propose that myelinating cells expressing PLP mutants alternate periods of active UPR, with subsequent attenuation of protein synthesis, and periods of inactive UPR and increased metabolism, leading to myelin production. In this view, cells switching off the UPR may be more vulnerable to pro-death signals due to the re-activation of the metabolism, such as calcium release or ROS production (Southwood et al., 2016). Further experiments performed at a single-cell level should be performed to confirm this model.

### ERQC in Krabbe disease

Krabbe disease (KD), also known as globoid cell leukodystrophy, is an autosomal recessive disorder characterized by rapid, fatal neurodegeneration associated with an extensive demyelination in both the CNS and PNS. Patients usually present an early-infantile phenotype in the first 6 months of life with irritability, spasticity, developmental delay, and progress to severe motor and mental deterioration that lead to death in childhood (Wenger et al., 2000). In addition to this early presentation, late-adult onset and milder variants exist, which progress more slowly (Wenger et al., 2000). Most of the KD hallmarks, such as severe demyelination, loss of oligodendrocytes, globoid cell infiltration and premature death are recapitulated in the twitcher (*twi*) mouse, a naturally occurring mutant (Kobayashi et al., 1980; Suzuki and Suzuki, 1983). All forms of KD are caused by mutations in the lysosomal enzyme galactosylceramidase (GALC) (Suzuki and Suzuki, 1970) that is essential for the hydrolysis of galactolipids, including galactosylsphingosine (psychosine) and galactosylceramide, a major lipid component of CNS and PNS myelin. In cells from KD patients, GALC activity is very low (0–5% of normal activity) and this leads to a progressive accumulation of psychosine, which is cytotoxic to oligodendrocytes and Schwann cells (Won et al., 2016). However, it should be noted that a recent work has questioned the relationship between psychosine and oligodendrocyte death in *twi* mice (Zhu et al., 2016). Over 100 deletion, frameshift and missense mutations in the *GALC* gene have been associated with KD (Won et al., 2016), but the correlation between *GALC* mutations and the clinical outcomes (age of onset or survival) is unclear and, even for the same mutation, a considerable clinical variability has been observed (Wenger et al., 2000). Similarly, measurement of GALC activity on whole-cell lysates does not reliably predict the severity of KD (Jardim et al., 1999). This difficulty to establish genotype-phenotype relationships is a major hurdle for current and future therapies against KD.

Newly synthesized GALC is co-translationally translocated into the ER and glycosylated in the Golgi apparatus (Spratley and Deane, 2016). GALC is then recognized by the mannose-6-phosphate receptor, which targets it to the lysosome either directly from the trans-Golgi network or indirectly via secretion and re-uptake (Nagano et al., 1998; Spratley and Deane, 2016). Once in the lysosome, GALC is cleaved into 50 and 30 KDa subunits and processes its lipid substrates. Mutations associated with KD were shown to strongly alter GALC trafficking (Table 1). The L629R mutation causes a severe lack-of-secretion phenotype and accumulation in the ER, whereas the GALC^I243T^ and GALC^D528N^ mutants are secreted into the medium but their reuptake is impaired (Lee et al., 2010). Moreover, the GALC^D528N^ mutant was found to be hyperglycosylated and misfolded and its function can be partially rescued by a treatment with the pharmacological chaperone α-lobeline (Lee et al., 2010). Another study confirmed that many GALC mutants were trapped within the ER instead of reaching the lysosome (Spratley et al., 2016). These data suggest that failure of ERQC is an important determinant in KD pathogenesis.

A recent work has proposed new mechanisms that may explain the considerable variability between KD patients (Shin et al., 2016). In this study, the authors show that cells expressing GALC mutants associated with infantile KD have no lysosomal GALC activity, whereas cells expressing late-onset mutations have a small residual activity in the lysosomes. Importantly, GALC trafficking to the lysosome is consistently impaired in infantile-onset mutants, but not (or to a lesser extent) in late-onset mutants. GALC activity is also detected in the ER and accounts for a substantial part of total GALC activity in the mutants, thus precluding total GALC activity to be a reliable marker of KD severity. The authors also suggest that three polymorphisms present in the *GALC* coding region (c.550T, c.742A, and c.1685C), which are not disease-causing *per se*, could modulate the impact of KD associated-mutations on GALC levels, activity and trafficking. In fact these additional amino acids changes in GALC sequence can further increase ER retention of KD mutants and/or reduce their activity, although the molecular bases of this process are currently unknown. The authors next propose a model for predicting KD severity: if patient-derived cells have less than 5% of normal GALC activity this indicates that the patient has KD. If the residual GALC activity is located in the lysosome, then the patient will have a late-onset disease with milder phenotype; if most of the remaining GALC activity is found in the ER or Golgi, then the patient will have an infantile-onset disease and a severe phenotype (Shin et al., 2016).

These results support the concept that impaired trafficking to the lysosome is an important feature for predicting the severity of KD and that boosting the ERQC by pharmacological chaperones could alleviate KD defects. Activation of the UPR has not been reported yet in KD.

### ER stress and vanishing white matter disease

Vanishing white matter disease (VWMD) is one of the most prevalent inherited childhood white matter disorders (Bugiani et al., 2010). VWMD is clinically characterized by slowly progressive cerebellar ataxia, spasticity and mild mental retardation that can worsen dramatically after episodes of stress, such as febrile infections, minor head trauma and acute fright (van der Knaap et al., 2006). The disease progresses rapidly and most patients die within few years after disease onset. Variation in disease severity is extremely wide: severe forms start during infancy and lead to premature death, while milder variants start in adolescence or adulthood and are characterized by slow disease progression (van der Knaap et al., 2006). VWMD is characterized by a severe deterioration of white matter, which shows diffuse vacuolation, myelin loss, presence of oligodendrocytes with abundant “foamy” cytoplasm and dysmorphic astrocytes (Bugiani et al., 2010). While apoptotic oligodendrocytes are observed in some affected areas, general oligodendrocytic density increases as a result of elevated pre-myelinating oligodendrocytes proliferation (Van Haren et al., 2004; Bugiani et al., 2011).

VWMD is caused by mutations in any of the five genes encoding the subunits of eukaryotic initiation factor 2B (eIF2B), which is pivotal for translation of mRNAs into proteins (van der Knaap et al., 2002). At least 120 different mutations have been described and approximately 90% are missense mutations (Bugiani et al., 2010). eIF2B serves as a guanine nucleotide exchange factor (GEF) of eIF2. The eIF2-GTP complex loads the initiator methionyl-tRNA (Met-tRNAi) to the ribosome upon recognition of the start codon of the mRNA. The eIF2-bound GTP is then hydrolyzed, with consequent release of eIF2 in its inactive GDP-bound form, which is ready for another round of translation initiation. As mentioned earlier, the α subunit of eIF2 is phosphorylated under various stress conditions. P-eIF2α converts eIF2 from a substrate of eIF2B to a strong competitor, thus reducing the formation of the eIF2-GTP complex and leading to a general attenuation of protein synthesis. As observed in patient-derived cells, almost all mutations associated with VWMD reduce the eIF2B GEF activity (Fogli et al., 2004; Horzinski et al., 2009). However, no correlation between residual GEF activity and disease severity has been found (Horzinski et al., 2009; Liu et al., 2011).

VWMD exclusively affects white matter of the brain, whereas gray matter and other organs are spared. One obvious question is why glial cells in the CNS are the most vulnerable to decreased eIF2B activity. The definitive answer is not known but, due to the central position of eIF2 in stress responses, it is likely that mutant eIF2B modulates the ability of oligodendrocytes to regulate protein synthesis in response to stress. Indeed, analysis of brain tissue from VWMD patients indicates that all three branches of the UPR are activated. Increased levels of XBP1s, BiP, CHOP, and GADD34 mRNAs are detected in brain autopsy samples from VWMD patients, as well as increased immunostaining for XBP1, ATF6, BiP, ATF4 and CHOP in oligodendrocytes and astrocytes (van der Voorn et al., 2005; van Kollenburg et al., 2006). Consistently, primary fibroblasts from VWMD patients are hypersensitive to ER-stress induced by a pharmacological agent (Kantor et al., 2005) and expression of VWMD-associated eIF2B mutants in oligodendroglial-derived cells upregulates ATF4, GADD34 and BiP even in the absence of a pharmacological stress agent (Kantor et al., 2008).

Several mouse models of VWMD have been generated in the last few years. Knock-in mice with authentic human R132H point mutation into *Eif2b5* gene display attenuated eIF2B activity in the brain and delayed development of white matter (Geva et al., 2010). However, these mutant mice do not exhibit most of the VWMD hallmarks, such as severe histological abnormalities, activation of the ER stress response and premature death (Geva et al., 2010; Marom et al., 2011), limiting their value for understanding KD pathogenesis. Using a completely different approach, Lin et al. have generated a mouse line that recapitulates most VWMD features. They have generated transgenic mice in which PERK signaling is specifically activated in oligodendrocytes in an inducible manner (Lin et al., 2013). Activation of PERK signaling in adult healthy animals is not detrimental to oligodendrocytes or myelin (Lin et al., 2013). In contrast, induction of PERK signaling in young animals (from P10) suppresses eIF2B activity and leads to a VWMD-like phenotype including severe hypomyelination, presence of foamy oligodendrocytes, animal trembling and death by P24 (Lin et al., 2014). These striking results demonstrate that chronic activation of PERK signaling in oligodendrocytes during myelin formation is pathogenic *per se*. In VWMD, it is plausible that eIF2B mutants have a lower translational activity that mimics a chronic phosphorylation of eIF2α and leads to a global attenuation of protein synthesis and/or a reprogramming of transcription by ATF4. Activation of this program in oligodendrocytes is likely not compatible with the production of the large amount of ER-synthesized proteins and lipids required for the myelination process. In this view, further inhibition of eIF2B by additional stresses (e.g., febrile infections) that increase eIF2α kinases activity (e.g., PKR) may exacerbate the VWMD phenotype. However it remains unclear how the reduced activity of eIF2B activates the IRE1 and ATF6 branches of the UPR as observed in VWMD patients (van der Voorn et al., 2005; van Kollenburg et al., 2006). Intriguingly, a recent study shows that cells expressing VWMD eIF2B mutants, despite having an enhanced ISR, do not tolerate an additional mutation preventing eIF2α phosphorylation (Sekine et al., 2016), although the mechanisms for this dependence are still unclear. This underlies that the link between VWMD and the UPR is extremely complex and needs further investigations.

Other data suggest that also astrocytes have a pivotal role in VWMD pathogenesis. Indeed, cultured astrocytes, but not oligodendrocytes derived from a VWMD patient, show defective development, aberrant morphology and antigenic phenotypes (Dietrich et al., 2005). Consistently, in oligodendrocytes/astrocytes co-cultures, it was observed that VWMD astrocytes, obtained from mice carrying R484W point mutation in *Eif2b4* gene, secrete factors that inhibit oligodendrocyte maturation, whereas wild-type astrocytes allow normal maturation of VWMD oligodendrocytes (Dooves et al., 2016). Altogether these results give the first evidence that eIF2B mutations impair differentiation and stress responses efficiency of myelinating and non-myelinating glial cells. The molecular bases of VWMD and in particular the role of the UPR in oligodendrocytes and astrocytes needs to be further explored.

## ERQC and charcot-marie-tooth (CMT) neuropathies

Inherited peripheral neuropathies (IPNs) are a group of disorders characterized by progressive degeneration of peripheral nerves. They have a frequency of 1:2500 with extensive clinical/genetic heterogeneity and no cure is currently available. The largest group of IPNs includes hereditary motor and sensory neuropathies (HMSN) or Charcot–Marie–Tooth (CMT) diseases, with the most severe forms also known as Dejerine-Sottas syndrome (DSS) and congenital hypomyelination (CH) (Baets et al., 2014; Jerath and Shy, 2015). At least 80 different genes have been linked to IPNs/CMTs, many of which are important for proper synthesis, structure and function of the myelin sheath (Brennan et al., 2015). Mutations in P0, PMP22 and Connexin32 (Cx32), the main structural proteins of PNS myelin, altogether represent the vast majority of all CMT cases (Jerath and Shy, 2015). Some Cx32 mutants display abnormal trafficking and do not reach the cell membrane, but accumulate in the ER/Golgi and undergo endosomal and proteasomal degradation (Deschênes et al., 1997; VanSlyke et al., 2000; Yum et al., 2002). However the impact of this retention on ERQC and its link to CMT pathogenesis has not been investigated yet (Kleopa et al., 2012). On the other hand, a number of mutations in P0 and PMP22 proteins have been extensively shown to generate misfolded molecules that exhaust the ERQC capacities (Figure 3 and Table 1). These aberrant molecules remain trapped in the ER, induce ER stress and eventually activate the UPR (D'Antonio et al., 2009; Lin and Popko, 2009; Clayton and Popko, 2016). The role of ERQC systems in this subset of ER stress-related neuropathies is discussed below.

### PMP22-CMT1A and CMT1E

PMP22 is a transmembrane glycoprotein that accounts for the 2–5% of the total myelin proteins in the PNS (Snipes et al., 1992; Li et al., 2013). It is encoded by the *PMP22* gene, and contains four putative transmembrane (TM) domains and one N-glycosylation site (Li et al., 2013). PMP22 protein is synthesized and modified in the ER/Golgi compartments and then delivered to myelin (Pareek et al., 1993, 1997; D'Urso et al., 1998). Interestingly, up to 70% of the newly synthesized PMP22 is rapidly degraded by the proteasome and only a very small amount reaches the cell surface (Figure 3A) (Pareek et al., 1993, 1997; Notterpek et al., 1999; Ryan et al., 2002).

Altogether, mutations in the human *PMP22* gene represent over 50% of all CMTs (Li et al., 2013). Duplication of the chromosomal region containing the *PMP22* gene is associated with the autosomal dominant demyelinating CMT1A neuropathy (Lupski et al., 1991), the most common form of CMT. Point mutations in *PMP22* sequence result either in hereditary neuropathy with liability to pressure palsy (HNPP) or in the demyelinating neuropathy known as CMT1E, which accounts for 1–5% of all CMTs (Li et al., 2013). The most extensively studied models of CMT1E and DSS are the Trembler (Tr) and Trembler-J (TrJ) mice, which carry the spontaneous G150D and L16P point mutations, respectively, also found in patients (Valentijn et al., 1992; Suter et al., 1992a,b).

These PMP22 mutants are misfolded proteins that appear to be stacked in the ER/Golgi and cytosolic compartments in cultured cellular systems, in mice and in human nerve biopsies (Naef et al., 1997; Colby et al., 2000; Hanemann et al., 2000; Ryan et al., 2002; Fortun et al., 2003; Shames et al., 2003). The mutant PMP22^TrJ^ protein engulfs the ERAD/proteasome system in Schwann cells, resulting in reduced proteasomal efficiency. The excess of PMP22^TrJ^ leads to the formation of aggresomes in the cytosol, that colocalize with heath shock protein (HSP) chaperones, proteasomes and autophagic markers (Hanemann et al., 2000; Ryan et al., 2002; Fortun et al., 2003, 2005). In TrJ mice, activated autophagy has been proposed to exert a relevant role in the clearance of these intracellular aggregates (Figure 3A and Table 1; Fortun et al., 2003, 2007). Aggresomes have also been observed upon PMP22 mutations other than L16P and G150D and in a model of CMT1A with overexpression of wild-type PMP22 due to 7 extra-copies of the gene (Shames et al., 2003; Liu et al., 2004; Fortun et al., 2006). However, in CMT1A patients that carry only three copies of the wild-type gene, PMP22 aggresomes were not detected (Hanemann et al., 2000). Recently, Hara and colleagues further explored the mechanism through which the mutant PMP22^TrJ^ and PMP22^Tr^ are retained in the ER and are polyubiquitinated (Hara et al., 2014). The authors found that both the CNX-dependent ERQC and the Rer1-mediated Golgi quality control systems are involved in the retention of PMP22^TrJ^, but not PMP22^Tr^ protein, despite all PMP22 variants (including the wild-type) interact with both Rer1 and CNX (Dickson et al., 2002; Hara et al., 2014). Furthermore, different ubiquitinating enzymes appear to mediate the polyubiquitination and degradation of the two mutants. While the ubquitin-ligase HRD1 is required for both molecules, gp78 is more specific for the PMP22^Tr^ form (Hara et al., 2014). Hence, this work elegantly underlies how different mutations in the very same molecule can engage different ERQC players to be disposed. Since, in parallel to autophagy, the ERAD pathway is fundamental for the clearance of the PMP22 mutants, the enhancement of ERAD might mitigate their cytotoxic effects (Hara et al., 2014). Curiously, even though both PMP22^TrJ^ and PMP22^Tr^ variants are ER retained, evidence for UPR activation has been so far reported only in TrJ mice, but not in Tr animals (Giambonini-Brugnoli et al., 2005; Okamoto et al., 2013). This suggests that the role of the UPR in CMT1E needs to be further investigated.

### P0–CMT1B

A member of the immunoglobulin-like superfamily, P0 (28–30 KDa) is codified as a type I transmembrane protein from the *MPZ* gene (D'Urso et al., 1990; You et al., 1991; Hayasaka et al., 1993). Abundantly and specifically expressed by myelinating Schwann cells, P0 protein alone represents 20–50% of all PNS myelin proteins (Everly et al., 1973; Greenfield et al., 1973; Patzig et al., 2011). Its main, but not exclusive, functions are to promote myelin compaction during myelinogenesis and to preserve myelin stability during adulthood, acting as an adhesion molecule (D'Urso et al., 1990, 1999; Filbin et al., 1990; Giese et al., 1992; Martini et al., 1995). Like PMP22, P0 is synthesized and processed in the ER/Golgi compartments, sorted into specific vesicles and transported to compact myelin (Figure 3B; Trapp et al., 1981, 1995; Brunden, 1992). The mature P0 protein is *N*-glycosylated and carries a stabilizing disulfide bond, together with other post-translational modifications (Eichberg, 2002).

Over 200 mutations in the human *MPZ* gene are known to cause up to 10% of all CMT cases. Different types of mutations, even those affecting the very same amino acid, result in different neuropathies, ranging from dys/demyelinating CMT1B/DSS/CH diseases to axonal CMT2 (Shy, 2006; Timmerman et al., 2014; Sanmaneechai et al., 2015). A subset of P0 mutations associated with altered P0 trafficking have been described, among which the best characterized are the R98C and S63del mutations in the extracellular domain of P0 (P0-ECD) (Figure 3B and Table 1; Shames et al., 2003; Khajavi et al., 2005; Wrabetz et al., 2006; Grandis et al., 2008; Prada et al., 2012; Saporta et al., 2012). In humans the R98C mutation results in severe early onset dysmyelinating and demyelinating neuropathy, partially recapitulated in R98C knock-in mice (Gabreëls-Festen et al., 1996; Bai et al., 2006; Saporta et al., 2012). The S63del mutation instead is associated with a milder form of CMT1B characterized by thinner myelin and late-onset demyelination, reproduced in S63del transgenic mice (Kulkens et al., 1993; Gabreëls-Festen et al., 1996; Wrabetz et al., 2006; Miller et al., 2012). Both these mutations cause their corresponding disease in heterozygous state in humans and either heterozygous (R98C) or hemizygous (S63del) state in mice, mimicking the dominant inheritance and underling a gain of toxic function mechanism. Indeed, both the P0^R98C^ and the P0^S63del^ proteins are misfolded and retained in the ER, where they cause ER stress and trigger a canonical UPR (Wrabetz et al., 2006; Pennuto et al., 2008; Miller et al., 2012; Saporta et al., 2012; D'Antonio et al., 2013). Altered polarity of the β-strand C in the P0-ECD carrying the S63del mutation is thought to be the structural aberration responsible for ER retention and UPR activation, likely because it promotes P0^S63del^ binding to BiP (Wrabetz et al., 2006). In addition, the mutant P0^S63del^ protein has also been proposed to exert a dominant negative effect on the wild-type P0 (P0^WT^), by partially trapping it into the ER and reducing its myelin levels (Figure 3B; Fratta et al., 2011). In both S63del and R98C mice, the activated UPR induces the expression of the pro-apoptotic factor CHOP. Intriguingly, the genetic ablation of CHOP ameliorates the neuropathic phenotype in S63del mice, whereas it has no effects in R98C animals (Pennuto et al., 2008; Saporta et al., 2012). What causes this difference? The main clinical feature of R98C mice is severe developmental hypomyelination without significant demyelination, whereas S63del mice display a milder hypomyelination followed by demyelination. The ablation of CHOP in S63del mice rescues demyelination and motor defects, but not hypomyelination, which may partially explain the lack of effects in R98C mice. Importantly, despite high levels of CHOP, neither S63del nor R98C nerves show significantly increased apoptosis, suggesting that CHOP may exert a different function in Schwann cells (Pennuto et al., 2008; Saporta et al., 2012). For example, in S63del mice the detrimental role of CHOP appears to be related to the upregulation of GADD34, that drives the dephosphorylation of P-eIF2α and restarts protein translation under a chronic ER stress (D'Antonio et al., 2013). In line with this, prolonging the attenuation of protein synthesis by genetic or pharmacological inhibition of GADD34 alleviates the S63del-CMT1B disease more efficiently than CHOP ablation, with improved hypomyelination, demyelination, motor performance and neurophysiological parameters (D'Antonio et al., 2013; Das et al., 2015). Whether GADD34 inhibition and prolonged eIF2α phosphorylation may improve also the R98C neuropathy is an interesting hypothesis that deserves testing. Surprisingly, and opposite to what was expected, the ablation of PERK in S63del nerves ameliorates, rather than aggravates, the neuropathy phenotype, despite decreased levels of P-eIF2α. This raises the intriguing possibility that PERK might have other effects on the neuropathy, uncoupled from the UPR and the attenuation of protein translation (Musner et al., 2016; Sidoli et al., 2016). It should also be noted that, despite the sustained ER stress, S63del Schwann cells manage to myelinate (albeit to a lower extent) and survive, indicating that significant UPR-related adaptive mechanisms are also in place to counter protein toxicity. Differently from what was observed in TrJ mice, in S63del mice neither cytoplasmic aggresomes nor autophagy genes induction have been detected, whereas ER chaperones and ERAD genes are strongly induced (Pennuto et al., 2008; D'Antonio et al., 2013). A better understanding of how these ERQC systems intervene in the recognition, folding and clearance of the mutant P0 will be of great relevance for the design of effective therapies. Curiously, despite CHOP, GADD34, and PERK clearly modulate disease severity in the S63del-CMT1B neuropathy, they do not appear to exert any essential role in physiological PNS myelination. In fact, upon ablation of these UPR mediators wild-type nerves develop normally and do not display myelin defects (Pennuto et al., 2008; D'Antonio et al., 2013; Musner et al., 2016; Sidoli et al., 2016), suggesting that they exert a pivotal role only when Schwann cells are already under stress.

### Treatments targeting the ERQC in myelinating disorders

All myelin disorders described above are caused by mutations leading to protein misfolding, impaired protein trafficking and/or activation of ER stress/UPR (summarized in Table 1). In all these diseases, the basal ERQC systems can be overwhelmed and the adaptive stress responses may become insufficient to prevent pathogenesis. In recent years, numerous studies have shown that enhancing ERQC pathways and the folding/degradation of proteins may provide a feasible and efficient therapeutic strategy in conformational diseases.

Pharmacological treatments boosting the UPR/ISR by inhibiting GADD34 have been used successfully in various preclinical models of neurological disorders (Hetz et al., 2013). For example, salubrinal restores motor function and ameliorates morphological and electrophysiological parameters in S63del mice (D'Antonio et al., 2013). Targeting the same pathway is also beneficial in a model of multiple sclerosis. Enhancement of PERK signaling by a genetic approach or sustained eIF2α phosphorylation with the GADD34 inhibitor guanabenz protects oligodendrocytes from death induced by EAE (Tsaytler et al., 2011; Lin et al., 2013; Way et al., 2015). Recently, Sephin1, a more specific GADD34 inhibitor, has been shown to largely prevent the motor, morphological, and molecular defects of S63del mice (Das et al., 2015). Finally, Sephin1 treatment has also been applied with success in the mutant SOD1^G93A^ mouse model of amyotrophic lateral sclerosis (ALS), a motor neuron disease. In this model, Sephin1 administration attenuates motor deficits and motor neuron loss, decreases the aggregation of mutant SOD1 and the level of ER stress (Das et al., 2015).

Pharmacological chaperone therapy (PCT) is another emerging approach that may be used to enhance ERQC in protein misfolding diseases. It is based on the administration of exogenous small-molecules that selectively bind mutant proteins and rescue their function by preventing aggregation and degradation and by promoting their trafficking. In lysosomal storage disorders, PCT clinical trials have produced encouraging results as a mono-therapy or in combination therapy (Parenti et al., 2015). PCT has not been tested yet in patients with inherited demyelinating disorders, however several studies highlight the potential of this approach. Treatment of KD cells with the chemical chaperone 4-phenylbutyric acid (4-PBA) increases the level and activity of GALC and promotes the trafficking of GALC mutants to the lysosomes (Shin et al., 2016). In another cell model of KD, treatment with N-octyl-4-epi-valienamine (NOEV) significantly increases the enzymatic activity and the processing of late-onset GALC mutants (Hossain et al., 2015). Moreover, administration of curcumin, a small molecule derived from the spice turmeric, known to correct the misfolding defects in models of cystic fibrosis (Egan et al., 2004), lowers mutant protein retention and the expression of UPR markers, and partially alleviates disease severity of *in vitro* and *in vivo* models of CMT1B and CMT1E (Khajavi et al., 2005, 2007; Patzkó et al., 2012; Okamoto et al., 2013). Curcumin therapy also leads to a modest amelioration of the phenotype in *msd* and PLP1 transgenic mouse models of PMD (Yu et al., 2012; Epplen et al., 2015), although the molecular mechanisms underlying curcumin action are still largely unknown.

### Concluding remarks

The accurate control of proper protein folding, maturation and trafficking is emerging as a key determinant in the physiology of myelinating glia. Several dysmyelinating disorders in both the CNS and the PNS show an important involvement of ER protein quality control systems in disease pathogenesis. However, each disease (and often each mutation) presents a unique signature in terms of ERQC pathways activation and mutant protein disposal (Table 1). A better understanding of the importance of each of these stress signaling pathways in physiological myelination and in disease will be crucial for the rational design of therapeutic strategies aimed at curing these often devastating conditions.

## Author contributions

VV, TT, and MD wrote the manuscript. VV prepared the figures. VV and TT prepared the Table. VV and TT contributed equally to this work.

## Funding

The laboratory is supported by grants from Telethon (GGP14147), the Italian Ministry of Health (GR-2011- 02346791) and AFM-Telethon (19498).

### Conflict of interest statement

The authors declare that the research was conducted in the absence of any commercial or financial relationships that could be construed as a potential conflict of interest.

## Abbreviations

ATF: Activating Transcription Factor
BiP: Immunoglobulin Heavy Chain-Binding Protein
CH: Congenital Hypomyelination
CHOP: C/EBP-Homologous Protein
CMT: Charcot-Marie-Tooth
CNS: Central Nervous System
CNX: Calnexin
CRT: Calreticulin
Derlin: Degradation In Endoplasmic Reticulum
DSS: Dejerine-Sottas Syndrome
EAE: Experimental Autoimmune Encephalomyelitis
ECD: Extracellular domain
eIF2: Eukaryotic Initiation Factor 2
ER: Endoplasmic Reticulum
ERAD: ER-Associated Degradation
ERQC: Endoplasmic Reticulum Quality Control
GADD34: Growth Arrest and DNA damage-inducible protein 34
GALC: Galactosylceramidase
GEF: Guanine nucleotide Exchange Factor
GRP94/78: Glucose Regulated Protein 94/78
HMSN: Hereditary Motor and Sensory Neuropathies
HNPP: Hereditary Neuropathy with liability to Pressure Palsy
HRD1: HMG-CoA Reductase Degradation protein 1
HSP: Heat Shock Protein
IPN: Inherited Peripheral Neuropathies
IRE1: Inositol Requiring Enzyme 1
ISR: Integrated Stress Response
KD: Krabbe disease
MAG: Myelin Associated Glycoprotein
MOG: Myelin Oligodendrocytes Glycoprotein
P0 or MPZ: Myelin Protein Zero
PCT: Pharmacological Chaperone Therapy
PERK: Protein-kinase RNA-like Endoplasmic Reticulum Kinase
PLP: Proteolipid Protein
PMD: Pelizaeus-Merzbacher Disease
PMP22: Peripheral Myelin Protein 22
PNS: Peripheral Nervous System
Rer1: Retention in Endoplasmic Reticulum Sorting Receptor 1
RIDD: Regulated IRE1-Dependent Decay
Sel1L: Suppressor/Enhancer of Lin-12-like
S1P/S2P: Site 1 Protease/Site 2 Protease
UPR: Unfolded Protein Response
VCP/P97: Valosin Containing Protein
VWMD: Vanishing White Matter Disease
XBP1: X-Box-Binding Protein 1.
